# A simple and clinically applicable model to predict liver-related morbidity after hepatic resection for hepatocellular carcinoma

**DOI:** 10.1371/journal.pone.0241808

**Published:** 2020-11-05

**Authors:** Jonggi Choi, So-Hyun Kim, Seungbong Han, Danbi Lee, Ju Hyun Shim, Young-Suk Lim, Han Chu Lee, Young-Hwa Chung, Yung Sang Lee, Sung-Gyu Lee, Ki-Hun Kim, Kang Mo Kim

**Affiliations:** 1 Department of Gastroenterology, Asan Liver Center, Asan Medical Center, University of Ulsan College of Medicine, Seoul, Republic of Korea; 2 Department of Applied Statistics, Gachon University, Seongnam-si, Republic of Korea; 3 Division of Hepatobiliary Surgery and Liver Transplantation, Department of Surgery, Asan Medical Center, University of Ulsan College of Medicine, Seoul, Republic of Korea; Indiana University, UNITED STATES

## Abstract

**Background & aim:**

Hepatic resection is a treatment option for patients with hepatocellular carcinoma (HCC). However, factors associated with candidacy for resection and predictive of liver-related morbidity after resection for HCC remain unclear. This study aimed to assess candidacy for liver resection in patients with HCC and to design a model predictive of liver-related morbidity after resection.

**Methods:**

A retrospective analysis of 1,565 patients who underwent liver resection for HCC between January 2016 and December 2017 was performed. The primary outcome was liver-related morbidity, including post-hepatectomy biochemical dysfunction (PHBD), ascites, hepatic encephalopathy, rescue liver transplantation, and death from any cause within 90 days. PHBD was defined as international normalized ratio (INR) > 1.5 or hyperbilirubinemia (> 2.9 mg/dL) on postoperative day ≥ 5.

**Results:**

The 1,565 patients included 1,258 (80.4%) males and 307 (19.6%) females with a mean age of 58.3 years. Of these patients, 646 (41.3%) and 919 (58.7%) patients underwent major and minor liver resection, respectively. Liver-related morbidity was observed in 133 (8.5%) patients, including 77 and 56 patients who underwent major and minor resection, respectively. A total of 83 (5.3%) patients developed PHBD. Multivariate analysis identified cut-off values of the platelet count, serum albumin concentration, and ICG R15 value for predicting liver-related morbidity after resection. A model predicting postoperative liver-related morbidity was developed, which included seven factors: male sex, age ≥ 55 years, ICG R15 value ≥ 15%, major resection, platelet count < 150,000/mm^3^, serum albumin concentration < 3.5 g/dL, and INR > 1.1.

**Conclusion:**

Hepatic resection for HCC was safe with 90-day liver-related morbidity and mortality rates of 8.5% and 0.8%, respectively. The developed point-based scoring system with seven factors could allow the prediction of the risk of liver-related morbidity after resection for HCC.

## Introduction

Advances in surgical techniques and improvements in perioperative care have expanded the indications for liver resection in patients with hepatocellular carcinoma (HCC) as well as reduced perioperative morbidity and mortality rates after liver resection [1, 2]. Furthermore, advances in liver resection have allowed more patients with HCC, particularly those with cirrhosis, to undergo this procedure and be potentially cured [3]. Although many models have been proposed to predict prognosis after liver resection, the designs, patient populations, and indications for liver resection have varied considerably among studies [1, 4–9]. Some models have included arbitrary cut-off points for parameters predictive of morbidity and mortality after liver resection [5, 7]. For example, the Child-Pugh score, a historic scoring system for grading the severity of cirrhosis, has been found to be appropriate for evaluating patients with cirrhosis; however, it may not be applicable to patients awaiting liver resection for HCC due to its lack of discriminative ability for patients with compensated cirrhosis [10]. In addition, the postoperative Child-Pugh score may not be predictive of patient outcomes [10]. Therefore, patient characteristics associated with favorable outcomes and overall eligibility for liver resection remain unclear. The present study determined whether patients would benefit from liver resection for HCC and attempted to identify factors predictive of liver-related morbidity after resection. In addition, these factors were used to design a model predictive of liver-related morbidity after liver resection for HCC.

## Methods

### Patient demographics and preoperative assessment

This study retrospectively analyzed 1,565 patients who underwent liver resection for HCC at Asan Medical Center, a tertiary care center in Seoul, Republic of Korea, between January 2016 and December 2017. All included patients were anonymized, and the demographic characteristics and underlying medical history of these patients were manually abstracted from their electronic medical records. All patients had been preoperatively diagnosed as having HCC by histological or radiologic examination according to international guidelines [3, 11], and all of them underwent thorough preoperative physical and laboratory examinations. Patients were selected for liver resection according to international treatment guidelines [3, 12] based on the medical condition of each patient. Medical condition was determined by preoperative liver function tests and imaging methods, including computed tomography (CT) and magnetic resonance imaging (MRI). Preoperative indocyanine green (ICG) clearance tests were performed to assess the maximum extent of resection of the liver parenchyma associated with a good functioning remnant liver [13]. At the discretion of each surgeon, patients with an anticipated small liver parenchyma remnant underwent preoperative portal vein embolization (PVE) to allow the future liver remnant to initiate hypertrophy [14]. All patients were evaluated preoperatively by anesthesiologists using the American Society of Anesthesiologists score [15]. This study was approved and was exempted from obtaining consent by the institutional review board of Asan Medical Center, Seoul, Republic of Korea (IRB Number. 2018–0839).

### Surgery, definitions of liver resection, postoperative monitoring

Of the 1,565 patients, 1,377 (88.0%) patients underwent conventional liver resection through an abdominal incision, and 188 (12.0%) patients underwent laparoscopic liver resection [16]. The Brisbane 2000 Terminology of Liver Anatomy and Resections was used [17]. Major hepatectomy was defined as a resection of three or more contiguous or noncontiguous liver segments [18]. Patients were monitored postoperatively in the recovery room and returned to the general ward unless they required special care in the ICU. Transfusion of fresh frozen plasma (FFP) was avoided for patients not requiring perioperative transfusion or coagulopathy. As a result, only 5 (0.3%) of the 1,565 patients received FFP transfusions. Blood samples were obtained daily for the first few postoperative days to assess liver function. Surgery-related complications were defined as described previously [19].

### Definition of post-hepatectomy biochemical dysfunction (PHBD) and liver-related morbidity

The primary outcome of this study was post-hepatectomy liver-related morbidity, which included post-hepatectomy liver failure, ascites requiring diuretics or invasive drainage procedures, hepatic encephalopathy, rescue liver transplantation, and death from any cause within 90 days after hepatectomy. PHBD was defined as an increased international normalized ratio (INR) (≥ 1.5) or hyperbilirubinemia (total bilirubin concentration ≥ 2.9 mg/dL) on or after postoperative day (POD) 5 [2, 20]. If the INR or bilirubin level was increased preoperatively, PHBD was defined as a higher INR or serum bilirubin level on or after POD 5 compared with the previous day [2]. Hyperbilirubinemia resulting from obvious causes, such as biliary obstruction, was not regarded as PHBD. In addition, in-hospital mortality and death from any cause within 30 and 90 days after the index operation were recorded.

### Statistical analysis

Continuous variables were compared using Student’s *t*-tests, and categorical variables were compared by Chi-square test or Fisher’s exact test as appropriate. To measure the ability of each variable to predict liver-related morbidity, the area under the receiver operator characteristic (AUROC) curve was calculated, with the cut-off value defined as that resulting in the maximization of the sum of sensitivity and specificity. For the development and validation of the prediction model, the 1,565 patients were randomly divided into two cohorts: a training cohort (n = 1,174; 75%) and a validation cohort (n = 391; 25%). A prediction model was designed based on the training dataset and applied to the validation dataset (S1 Methods). The baseline characteristics of the patients in these two cohorts are presented in Table 1. Liver morbidity was modeled by fitting a logistic (base) model and converting the proposed model into a scoring point system for easy interpretation [21]. Factors associated with patient demographics, disease severity, health status, and comorbidities evaluated as prognostic variables are presented in Table 1, with the best prognostic variables selected using a bootstrap resampling approach. A total of 1000 bootstrap samples were generated, followed by a backward stepwise variable selection procedure based on the Akaike information criterion for each bootstrap sample. The number of candidate variables remaining in the final model among the 1,000 replications was counted, with variables appearing > 850 times included in the final scoring model. Clinical importance was also considered in choosing variables that were both clinically meaningful and statistically significant. Variables selected for the final model were gender, presence of cirrhosis, INR, albumin concentration, planned type of resection, and platelet count. Both discriminative and calibration abilities were examined according to the AUROC curves and calibration curves of the training and validation cohorts. Calibration ability was determined by the Hosmer-Lemeshow test. To handle missing data, the single imputation method was applied based on the mice package in R [22]. All data analyses were performed using R software (version 3.5.2; R Foundation for Statistical Computing, Vienna, Austria). All reported *P* values are two-sided, and *P* values < 0.05 were considered statistically significant.

**Table 1:** Baseline characteristics of the patients included in this study.

Characteristics	All (n = 1,565)	Training cohort (n = 1,174)	Validation cohort (n = 391)	*P*
Age, years	58.3 ± 9.9	58.4 ± 10.0	57.9 ± 9.4	0.46
Gender, male/female, n (%)	1258/307 (80.4/19.6)	932/242 (79.4/20.6)	326/65 (83.4/16.6)	0.10
Etiologies, n (%)				0.71
Alcohol	101 (6.5)	24 (6.1)	77 (6.6)	
HBV	1,265 (80.8)	325 (83.1)	940 (80.1)	
HCV	52 (3.3)	10 (2.6)	42 (3.6)	
HBV + HCV	9 (0.6)	2 (0.5)	7 (0.6)	
NBNC	138 (8.8)	30 (7.7)	108 (9.2)	
ASA fitness grade, n (%)				0.15
1	30 (1.9)	7 (1.8)	23 (2.0)	
2	1,385 (88.5)	335 (85.7)	1,050 (89.4)	
3	146 (9.3)	48 (12.3)	98 (8.3)	
4	4 (0.3)	1 (0.3)	3 (0.3)	
Body mass index, kg/m^2^	24.2 ± 3.1	24.1 ± 3.0	24.2 ± 3.1	0.65
Comorbidities, n (%)				
Diabetes	314 (20.1)	76 (19.4)	238 (20.3)	0.78
Hypertension	546 (34.9)	147 (37.6)	399 (34.0)	0.22
Cardiovascular	19 (1.2)	5 (1.3)	14 (1.2)	0.99
Renal	13 (0.8)	3 (0.8)	10 (0.9)	0.99
Oncologic	30 (1.9)	9 (2.3)	21 (1.8)	0.67
Respiratory	23 (1.5)	6 (1.5)	17 (1.4)	0.99
Cirrhosis, n (%)	603 (38.5)	168 (43.0)	435 (37.1)	0.60
Varices, n (%)	13 (0.8)	1 (0.3)	12 (1.0)	0.26
Ascites, n (%)	1 (0.1)	0 (0.0)	1 (0.1)	0.99
MELD score	7.7 ± 1.4	7.7 ± 1.5	7.7 ± 1.4	0.73
Child-Pugh score				
5	1,067 (68.2)	282 (72.1)	785 (66.9)	0.06
6	105 (29.3)	105 (26.9)	353 (30.1)	
≥ 7	40 (2.6)	4 (1.0)	36 (3.0)	
Previous TACE, n (%)	258 (16.5)	56 (14.3)	202 (17.2)	0.21
Previous PVE, n (%)	156 (10.0)	38 (9.7)	118 (10.1)	0.93
Baseline laboratory exam				
Hemoglobin, g/dL	13.6 ± 1.6	13.7 ± 1.7	13.6 ± 1.6	0.09
Platelets, ×1,000/mm^3^	178.2 ± 73.8	177.9 ± 84.1	178.3 ± 70.0	0.93
Prothrombin time, INR	1.1 ± 0.1	1.1 ± 0.1	1.1 ± 0.1	0.90
Creatinine, mg/dL	0.9 ± 0.4	0.9 ± 0.6	0.9 ± 0.3	0.23
Albumin, g/dL	3.7 ± 0.4	3.7 ± 0.4	3.7 ± 0.4	0.43
AST, IU/L	37.8 ± 31.8	37.6 ± 32.3	37.8 ± 31.6	0.90
ALT, IU/L	35.0 ± 36.4	35.5 ± 33.8	34.9 ± 37.3	0.77
Total bilirubin, mg/dL	0.6 ± 0.4	0.6 ± 0.3	0.7 ± 0.4	0.11
Sodium, mmol/L	139.9 ± 2.5	140.0 ± 2.5	139.9 ± 2.5	0.44
Estimated GFR	92.7 ± 15.4	92.9 ± 16.0	92.7 ± 15.2	0.80
AFP, ng/mL, median [IQR]	10.8 [3.6–177.7]	12.2 [3.9–171.0]	10.6 [3.5–187.4]	0.73
ICG R15, median [IQR]	13.2 [10.3–16.6]	12.8 [10.3–16.8]	13.2 [10.2–16.6]	0.77
Operation				
Anesthesia time, min, median [IQR]	245 [210–300]	245 [210–300]	246 [210–300]	0.73
Operation time, min, median [IQR]	211 [175–263]	208 [171–262]	212 [177–263]	0.29

Abbreviations: AFP: alpha-fetoprotein, ALT: alanine aminotransferase, ASA: American Society of Anesthesiologists, AST: aspartate aminotransferase, GFR: glomerular filtration rate, HBV: hepatitis B virus, HCV: hepatitis C virus, ICG: indocyanine green, INR: international normalized ratio, IQR: interquartile range, NBNC: non-HBV and non-HCV, PVE: portal vein embolization, TACE: transarterial chemoembolization

## Results

### Patient characteristics

The baseline characteristics of the included patients are summarized in Table 1. The 1,565 patients who underwent liver resection for HCC included 1,258 (80.4%) males and 307 (19.6%) females with a mean age of 58.3 years (Table 1). The major etiology of the underlying liver disease was chronic HBV infection (80.8%). The most common comorbidities were hypertension (34.9%) and diabetes (20.1%), with 37.9% of patients having liver cirrhosis.

To construct a prediction model for post-hepatectomy liver-related morbidity, the entire study cohort was divided into the training and validation cohorts. There were no significant differences in baseline characteristics between the training and validation cohorts.

### Types of liver resection

Of the 1,565 patients, 646 (41.3%) and 919 (58.7%) patients underwent major and minor liver resection, respectively (S1 Table). Detailed examination showed that 319 (20.4%) and 155 (9.9%) patients underwent right and left hepatectomy, respectively, with 8 (0.6%) and 5 (0.2%) patients undergoing extended right and left hepatectomy, respectively. In addition, 147 (9.4%) patients underwent resection of more than two segments, a category not included in the standard definition of liver resection, and 272 (17.4%) and 85 (5.4%) patients underwent segmentectomy and bisegmentectomy, respectively (S2 Table).

### Liver-related morbidity and mortality

Liver-related morbidity occurred in 133 (8.5%) patients, including 77 and 56 patients who underwent major and minor resection, respectively. A total of 80 (5.1%) patients had a serum bilirubin concentration of ≥ 2.9 mg/dL or an INR of ≥ 1.5 on or after POD 5, defined as PHBD (Table 2).

**Table 2:** Morbidity and mortality rates after liver resection for hepatocellular carcinoma.

Morbidities	All (n = 1,565)	Major resection (n = 646)	Minor resection (n = 919)	*P*
Liver-related morbidities	133 (8.5%)	77 (11.9%)	56 (6.1%)	< 0.001
INR ≥ 1.5 on or after POD 5*	48 (3.1)	33 (5.3)	15 (1.8)	< 0.001
Total bilirubin ≥ 2.9 mg/dL on or after POD 5*	49 (3.1)	29 (4.6)	20 (2.3)	0.02
INR ≥ 1.5 or total bilirubin ≥ 2.9 mg/dL on or after POD 5*	80 (5.1)	50 (8.0)	30 (3.5)	< 0.001
Ascites	51 (3.3)	26 (4.0)	25 (2.7)	0.20
Encephalopathy	1 (0.1)	0 (0.0)	1 (0.1)	0.99
Liver transplantation due to liver failure	2 (0.1)	1 (0.2)	1 (0.1)	0.99
Mortality at 30 days	4 (0.3)	2 (0.3)	2 (0.2)	0.99
Mortality at 90 days	13 (0.8)	9 (1.4)	4 (0.4)	0.08
Medical morbidities				
ICU stay/mechanical ventilation	20 (1.3)	10 (1.5)	10 (1.1)	0.57
Sepsis/bacteremia	14 (0.9)	4 (0.6)	10 (1.1)	0.49
Pneumonia	7 (0.4)	2 (0.3)	5 (0.5)	0.76
Renal failure	3 (0.2)	1 (0.2)	2 (0.2)	0.99
Cardiovascular	3 (0.2)	1 (0.2)	2 (0.2)	0.99
Neurologic	4 (0.3)	2 (0.3)	2 (0.2)	0.99
Surgery-related morbidities				
Bleeding	7 (0.4)	4 (0.6)	3 (0.3)	0.64
Wound complications	47 (3.0)	20 (3.1)	27 (2.9)	0.98
Bile leak/perihepatic abscess	12 (0.8)	3 (0.5)	9 (1.0)	0.64
Fluid collection	60 (3.8)	31 (4.8)	29 (3.2)	0.13
Portal vein thrombosis	14 (0.9)	7 (1.1)	7 (0.8)	0.69
Pneumothorax	4 (0.3)	3 (0.3)	9 (1.0)	0.39
Any post-hepatectomy morbidity	206 (13.2)	89 (13.8)	95 (10.3)	0.045
Length of hospital stay, days	13 [11–16]	13 [11–17]	12 [10–16]	0.003
Length of hospital stay after resection, days	10 [9–12]	10 [9–12]	10 [9–12]	0.003

*Greater than on the previous day. Abbreviations: ICU: intensive care unit, INR: international normalized ratio, POD: postoperative day

Specifically, 48 (3.1%) and 7 (0.4%) patients showed INRs of ≥ 1.5 and ≥ 2.0, respectively, and 294 (18.8%) and 49 (3.1%) patients showed total bilirubin concentrations of ≥ 1.5 mg/dL and ≥ 2.9 mg/dL, respectively, on or after POD 5. A total of 51 (3.3%) patients had postoperative ascites requiring diuretics or invasive drainage procedures. In addition, 1 patient developed hepatic encephalopathy resulting from the deterioration of liver function, and 2 patients had liver failure and subsequently underwent rescue liver transplantation. A total of 13 patients died within 90 days after the operation, including 4 patients who died within 30 days (S3 and S4 Tables).

### Postoperative course and hospital stay

Of the 1,565 patients, 206 (13.2%) patients had some type of post-hepatectomy morbidity (Table 2). The most common surgery-related morbidity was fluid collection requiring invasive drainage procedures, which occurred in 60 (3.8%) patients. A total of 20 (1.3%) patients remained in the ICU for postoperative stabilization, and 14 (0.9%) patients had severe infection including bacteremia. The average length of hospital stay was 13.0 days (interquartile range [IQR]: 11.0–16.0 days), and the mean length of stay after surgery was 10.0 days (IQR: 9.0–12.0 days).

### Assessment of operative candidacy

Cirrhosis was significantly more common (51.0% vs. 36.8%; *P* < 0.001), and ICG R15 was significantly lower (14.1 ± 7.2 vs. 16.3 ± 11.2; *P* = 0.01) in patients with liver-related morbidity than in those without liver-related morbidity. The optimal cut-off value of ICG R15 for predicting liver-related morbidity in these patients was 16.3% (14.5% in patients who underwent major resection and 19.3% in patients who underwent minor resection) (S5 Table).

The optimal cut-off values of the platelet count for predicting liver-related morbidity in patients who underwent major and minor resection were 158,000/mm^3^ and 133,000/mm^3^, respectively. The liver-related morbidity rates of patients with platelet counts of > 150,000/mm^3^ and < 100,000/mm^3^ were 6.8% and 13.4%, respectively.

The optimal cut-off values of the serum albumin concentration for predicting liver-related morbidity in patients who underwent major and minor resection were 3.6 g/dL and 3.4 g/dL, respectively. The optimal cut-off value of the prothrombin time measured by INR was 1.12 (1.15 in patients who underwent major resection and 1.12 in patients who underwent minor resection).

### Construction of a risk prediction model

**Fig 1:**
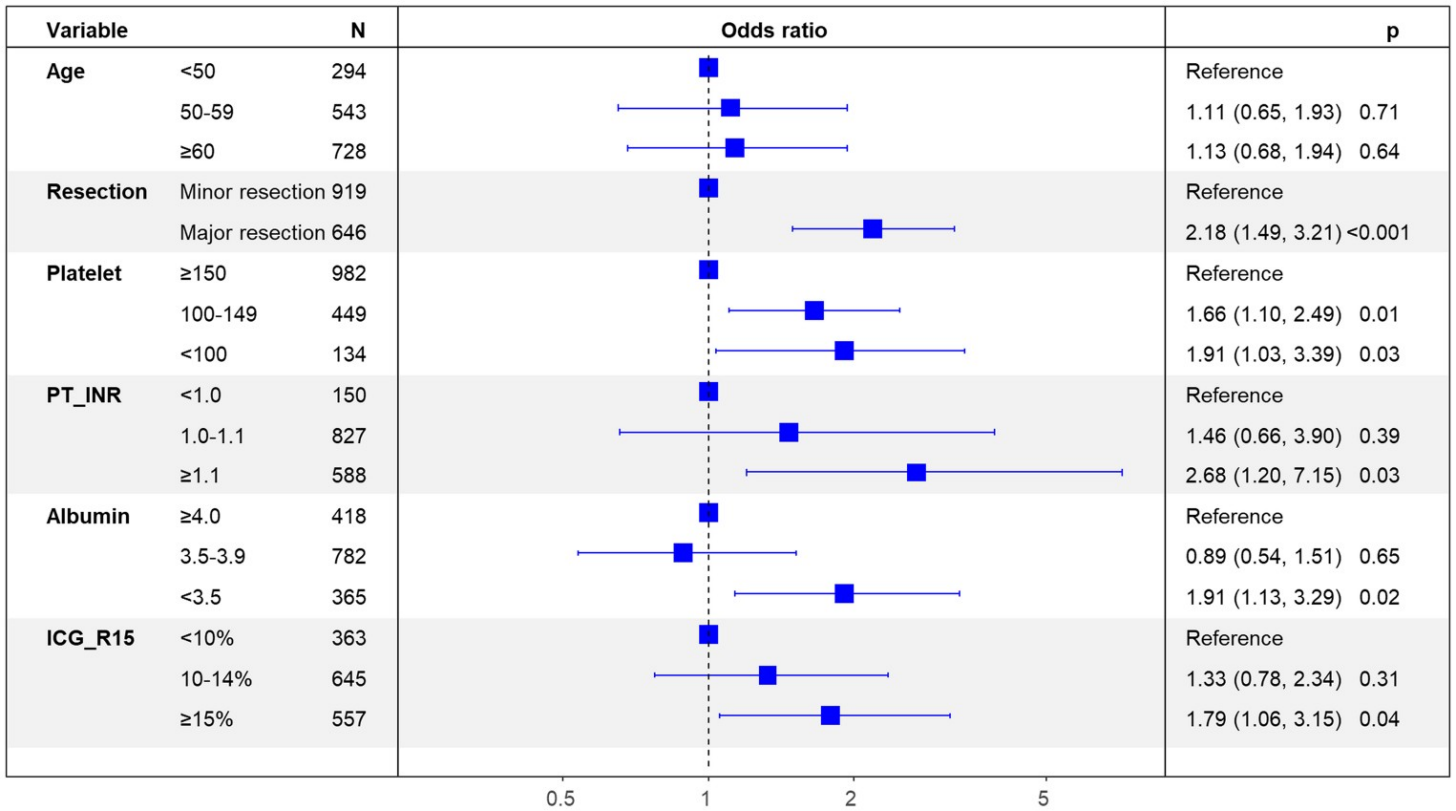
Forest plot of the results of multivariable analysis of factors independently associated with liver-related morbidity after liver resection for hepatocellular carcinoma.

Multivariable analysis identified major resection (adjusted odds ratio [AOR] 2.18; 95% confidence interval [CI]: 1.49–3.21; *P* < 0.001), platelet count < 150,000/mm^3^ (AOR: 1.91; 95% CI: 1.03–3.39; *P* = 0.03), INR ≥ 1.1 (AOR: 2.68; 95% CI: 1.20–7.15; *P* = 0.03), serum albumin concentration < 3.5 g/dL (AOR: 1.91; 95% CI: 1.13–3.29; *P* = 0.02), and ICG R15 value ≥ 15% (AOR: 1.79, 95% CI: 1.06–3.15; *P* = 0.04) as predictive factors of postoperative liver-related morbidity (Fig 1).

Logistic regression analysis of patients in the training cohort identified male sex, major resection, platelet count < 150,000/mm^3^, serum albumin concentration < 3.5 g/dL, INR > 1.1, and ICG R15 value ≥ 15 as factors for predicting postoperative liver-related morbidity. Table 3 shows the estimated regression coefficients in the logistic model. When transformed to an integer-based risk score, male sex, age ≥ 55 years, and ICG R15 value ≥ 15% were each assigned a score of 1 point, and major resection, platelet count < 150,000/mm^3^, serum albumin concentration < 3.5 g/dL, and INR > 1.1 were each assigned a score of 2 points (Table 3).

**Table 3:** Regression coefficient and odds ratio estimation from the training cohort and the corresponding risk scores.

	OR (95% CI)	B coefficient	*P*	Risk score
Sex				
Female	1.00			0
Male	1.70 (0.93–3.10)	0.5298	0.085	1
Age, years				
< 55	1.00			0
≥ 55	1.50 (0.94–2.37)	0.4030	0.087	1
Type of resection planned				
Minor	1.00			0
Major	2.24 (1.45–3.46)	0.8061	0.0003	2
Platelets, ×1000/mm^3^				
≥ 150	1.00			0
< 150	1.84 (1.20–2.83)	0.6099	0.005	2
Serum albumin, g/dL				
≥ 3.5	1.00			0
< 3.5	1.89 (1.21–2.95)	0.6382	0.005	2
Prothrombin time, INR				
≤ 1.1	1.00			0
> 1.1	2.67 (1.73–4.12)	0.9804	< 0.001	2
ICG R15				
< 15%	1.00			0
≥ 15%	1.55 (1.00–2.39)	0.4371	0.048	1

*Abbreviations: ICG: indocyanine green, INR: international normalized ratio, OR: odds ratio

**Fig 2:**
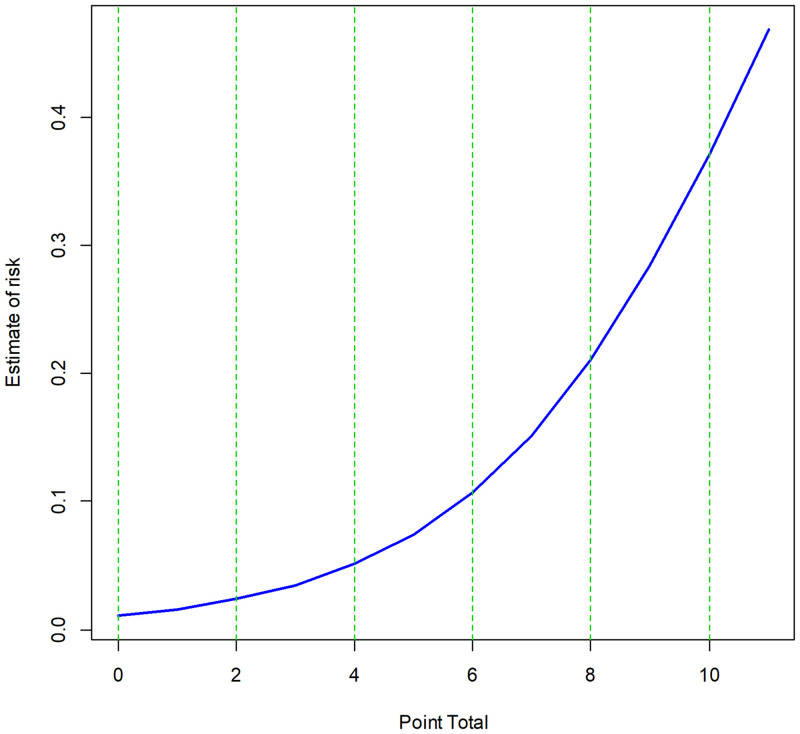
Calculated risk of post-hepatectomy liver-related morbidity as a function of risk score.

The sum of these seven scores reflects the estimated risk of liver-related morbidity after liver resection for HCC (Fig 2 and Table 4).

**Table 4:** Cumulative risk scores and corresponding risks of developing post-hepatectomy liver-related morbidity in patients who underwent liver resection for hepatocellular carcinoma.

Score group	Range of estimated risk	Score	Estimated risk
Low 0 − 2	1 − 2%	0	1.1%
		1	1.6%
		2	2.4%
Intermediate 3–5	3 − 7%	3	3.5%
		4	5.1%
		5	7.4%
High 6–8	11 − 21%	6	10.7%
		7	15.2%
		8	21.1%
Very high 9 − 11	29 − 47%	9	28.5%
		10	37.2%
		11	46.7%

**Fig 3:**
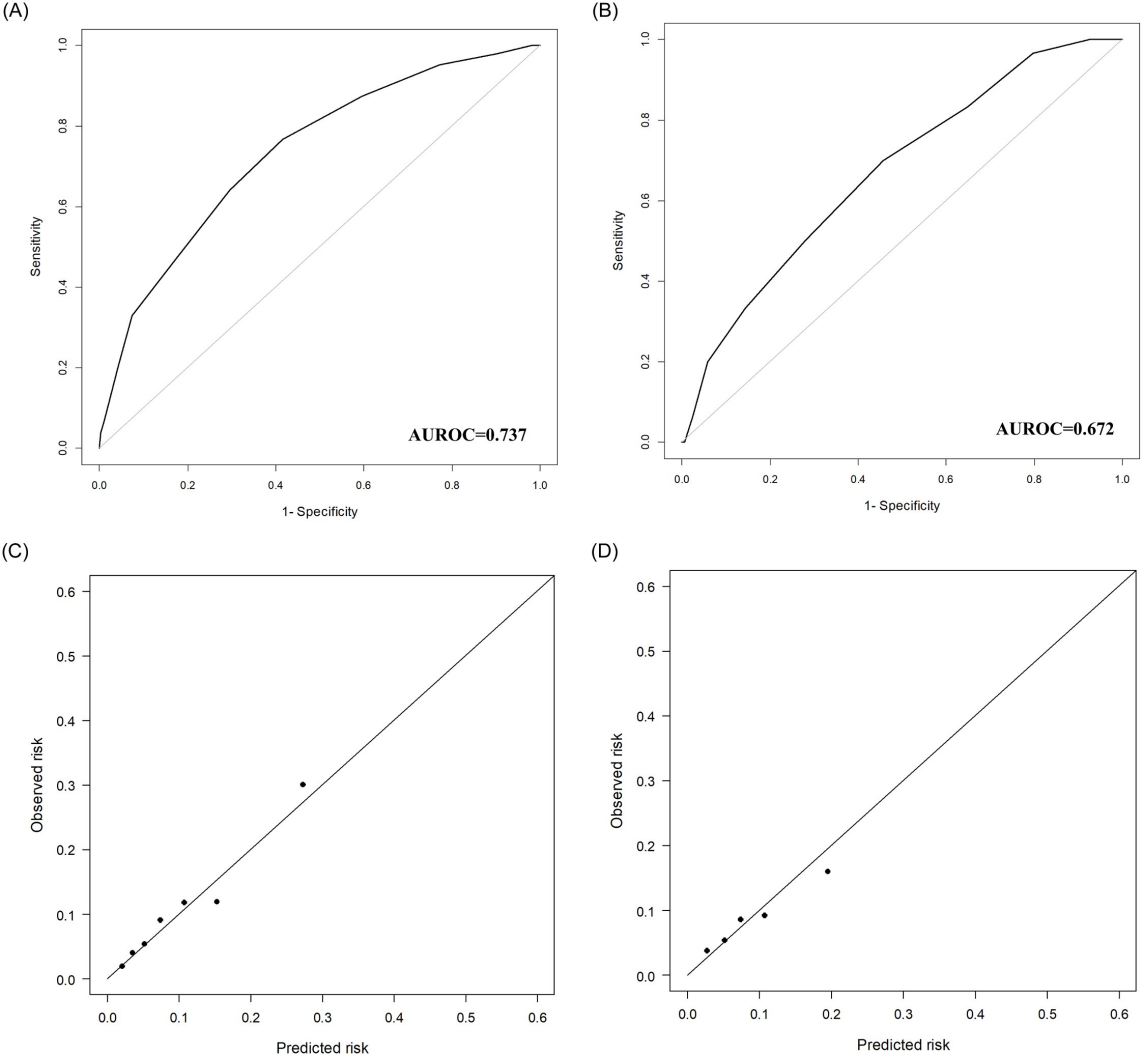
Receiver operating characteristic curves and calibration charts of the training and test cohorts. A. Receiver operating characteristic curve of the training cohort. B. Receiver operating characteristic curve of the test cohort. C. Calibration chart of the predicted versus observed risk of the training cohort. D. Calibration chart of the predicted versus observed risk of the test cohort.

Receiver operating characteristic (ROC) curves for predicting postoperative liver-related morbidity in the training and test cohorts are shown in Fig 3A and 3B, respectively. The AUROC curves of the training and test cohorts were 0.737 (95% CI: 0.687–0.787) and 0.672 (95% CI: 0.577–0.767), respectively (Fig 3A and 3B). The calibration curves shown in Fig 3C and 3D indicated good agreement between the risk predicted by the model and the observed risk. Hosmer-Lemeshow test results also showed adequate agreement in the training and test cohorts with *P* values of 0.965 and 0.726, respectively.

### Application of the predictive model

To test the point-based prediction model described above, it was applied to actual patients. For example, a 56-year-old man (2 points) was scheduled to undergo major liver resection (2 points) for HCC. Preoperative laboratory tests revealed a platelet count of 160,000/mm^3^ (0 points), a serum albumin concentration of 3.7 g/dL (0 points), a prothrombin time (INR) of 1.05 (0 points), and an ICG R15 value of 16% (1 point). Collectively, this patient had a score of 5 points, which corresponded to an estimated risk of 7.4% of liver-related morbidity after liver resection for HCC (Table 5).

**Table 5:** Representative scoring of risk factors in the model for a patient.

Score calculation		
Risk factor	Value	Score
Sex	Male	1
Age	56 years	1
Type of resection planned	Major resection	2
Platelets	160,000/mm^3^	0
Serum albumin	3.7 g/dL	0
Prothrombin time	1.05	0
ICG R15	16%	1
**Risk estimation**		
Total score	5	
Score group	Intermediate	
Estimated risk	7.4%	

## Discussion

The present study evaluated the demographic and preoperative and perioperative characteristics of 1,565 patients with HCC to determine whether they are candidates for liver resection and to identify factors predictive of liver-related morbidity after resection. Liver resection for HCC was safe with a liver-related morbidity rate of < 10% and a 90-day mortality rate of < 1%. Factors associated with liver-related morbidity included lower serum albumin concentration, thrombocytopenia, prolonged prothrombin time as measured by INR, and higher ICG R15 value. These factors were included in a point-based model to preoperatively predict liver-related morbidity after liver resection for HCC, which showed good discriminative ability and performance.

Most patients with HCC have underlying liver diseases, such as chronic hepatitis B, HCV infection, alcoholic liver disease, and cirrhosis of any cause, which are responsible for the development of HCC [23]. Therefore, these patients should not be regarded as similar to those undergoing liver resection for non-HCC causes without underlying liver diseases. The ability to preoperatively predict poorer outcomes after liver resection can help in the selection of eligible candidates for surgery to ensure optimal oncologic outcomes for patients with HCC. The present study showed that a combination of conventional liver function tests, including serum albumin concentration, INR, platelet count, and ICG R15 value, was significantly associated with outcomes after liver resection. These factors allowed the development of a point-based prediction model for estimating the risk of liver-related morbidity. This model can be easily applied in actual clinical settings.

Previous studies have proposed various risk models to predict mortality or post-hepatectomy liver failure after liver resection [1, 24–27]. A large-scale retrospective study of 2,056 patients who underwent liver resection developed a risk scoring system based on POD3 INR, bilirubin, and creatinine to predict the 90-day mortality after hepatectomy [1]. Similar to our scoring system, the study incorporated serum bilirubin and creatinine into a scoring model. However, we intended to develop a scoring system based on preoperative characteristics to predict post-hepatectomy liver-related morbidity before making a clinical decision. Therefore, we used only preoperative variables for constructing the prediction model in the present study. Another study compared the morbidity and mortality prediction performance after liver resection among the MELD score, 50–50 criteria, and post-hepatectomy liver failure, suggesting that the MELD score could be appropriate for the early prediction of morbidity [4]. In the present study, the MELD score was not an independent predictor of liver-related morbidity after liver resection; the mean MELD score was 7.7, and most of the included patients had a low MELD score (< 10). Generally, patients considering liver resection for the treatment of HCC have well-preserved liver function. Indeed, more than 90% of our patients had good liver function of Child-Pugh class A. As serum bilirubin concentration and INR are nearly normal in Child-Pugh class A, the MELD score would not have good discriminative ability.

Our scoring system is advantageous compared with previous scoring systems. First, our scoring system included patients with homogeneous indications for liver resection, i.e., HCC. Previous prediction models were derived from patients with heterogeneous indications for liver resection, such as colorectal cancer with liver metastasis and cholangiocarcinoma [4, 5, 7, 20]. The aim of our study was to preoperatively and effectively predict liver-related morbidity after liver resection for HCC. The selection of treatment modality for HCC should consider various factors including the risk of liver-related morbidity after liver resection, severity of the underlying liver disease, and patient demographics; thus, these factors were incorporated into our scoring system. Second, our scoring system was derived from more than 1,500 patients who underwent liver resection. In comparison with other scoring systems, the number of included patients was relatively large, allowing us to maximize the predictive ability of our scoring system [4, 20, 25]. Third, we only included preoperative variables in our scoring system because the purpose of our system was to guide the treatment decision for patients with HCC by accurately estimating the potential risk of liver-related morbidity after liver resection for HCC. In other words, a scoring system with postoperative variables might be less meaningful for this purpose. Lastly, our scoring system was designed with simple numeric points based on preoperative variables, which can be readily calculated in the clinic, and the risk estimated by this scoring system is easily interpreted.

The present study also had several limitations. First, we were unable to measure the residual liver volume preoperatively. Although no consensus has been reached regarding the remnant liver volume that is considered safe after liver resection, preoperative radiologic assessment of the residual liver volume indicated that it may be predictive of hepatic dysfunction after major liver resection [6, 28]. Second, our model was derived from a patient cohort in a single center and was not validated externally. However, this design excluded the effects of different operative procedures and postoperative care. Third, the statistical thresholds presented in the present study should not be regarded as rigid criteria for deciding whether a patient with HCC should undergo liver resection or should be managed non-operatively. Fourth, we included patients who underwent laparoscopic and open liver resection. Recently, laparoscopic liver resection is known to be associated with better outcomes such as lower morbidity after liver resection and better patient tolerability [29, 30]. However, only 12.0% of the included patients underwent laparoscopic liver resection in the present study, and no difference was observed in the risk of liver-related morbidity between those with open liver resection and those with laparoscopic liver resection. Therefore, a prediction model incorporating these two operational techniques should be further investigated in the future. Lastly, a recent study developed a nomogram by combining liver stiffness with conventional risk factors such as age and liver function to predict postoperative morbidity after liver resection for HCC [31]. Liver stiffness may represent the fibrotic burden of the liver parenchyma, which is strongly associated with liver function. Therefore, incorporating this measurement into the prediction model might provide additional benefit for the prediction of the risk of liver-related morbidity after liver resection. However, the measurement of liver stiffness was not a routine procedure for all patients planning to undergo liver resection for HCC in our center.

## Conclusions

In conclusion, this study describes the outcomes of 1,565 patients who underwent liver resection for HCC in a single tertiary center over a recent 2-year period. Liver resection was safely performed with a very low mortality rate and a low morbidity rate compared with those in previous studies. These results were used to design a point-based model predictive of liver-related morbidity. Male sex, age > 55 years, major resection, platelet count < 150,000/mm^3^, serum albumin concentration < 3.5 g/dL, INR > 1.1, and ICG R15 value ≥ 15% were risk factors for predicting postoperative liver-related morbidity. The sum of the scores for these seven factors could allow the prediction of the risk of liver-related morbidity after liver resection for HCC.

## Supporting information

S1 Methods Development and validation of the prediction model. (DOCX)

S1 Table Baseline characteristics of patients according to the type of resection. (DOCX)

S2 Table Details of the treatment of the 1,565 patients enrolled in this study. (DOCX)

S3 Table Summary of patients who died after liver resection. (DOCX)

S4 Table Comparison of the baseline characteristics of patients who did and did not die within 90 days after liver resection for hepatocellular carcinoma. (DOCX)

S5 Table Morbidity and mortality rates after liver resection according to baseline characteristics and resection type. (DOCX)

S1 Dataset (CSV)
