# Supplementary material for: A simple and clinically applicable model to predict liver-related morbidity after hepatic resection for hepatocellular carcinoma
Source: PLoS One. 2020 Nov 5;15(11):e0241808. doi: 10.1371/journal.pone.0241808 (PMC7643950; doi:10.1371/journal.pone.0241808)
Supplement: S1 Methods — (DOCX) [file pone.0241808.s001.docx]

**S1 Methods**

**Development and validation of the prediction model**

Variables selected for the final model were gender, presence of cirrhosis, INR, albumin concentration, planned type of resection, and platelet count. If the event number per variable (EPV) was 17, six prognostic factors would be adequate for the final prediction model.[1] To avoid a multicollinearity problem among the finally selected variables, the variation inflation factor (VIF) was calculated and multicollinearity was assessed (VIF ≥5). The assumption of linearity in the logistic regression model was evaluated by fitting the multiple fractional polynomial model.[2] No violations were found on the multicollinearity and linearity assumption tests. INR, albumin concentration, and platelet count were categorized for better interpretation. Their distributions in the training set and their clinical cut-off values were considered. The cut-off values were finally determined to be INR 1.0, 1.1, and 1.2; albumin concentration 3.5 mg/dL; and platelet count 150,000/mm^3^.

**References for Supporting Methods**

1. Peduzzi P, Concato J, Kemper E, et al (1996) A simulation study of the number of events per variable in logistic regression analysis. J Clin Epidemiol 49:1373-1379

2. Benner A Package ‘mfp’, The R Foundation, 2010;computer program.
