# Supplementary material for: A simple and clinically applicable model to predict liver-related morbidity after hepatic resection for hepatocellular carcinoma
Source: PLoS One. 2020 Nov 5;15(11):e0241808. doi: 10.1371/journal.pone.0241808 (PMC7643950; doi:10.1371/journal.pone.0241808)
Supplement: S1 Table — (DOCX) [file pone.0241808.s002.docx]

**S1 Table.** Baseline characteristics of the patients according to the type of resection

| **Characteristics** | **All**  **(n = 1,565)** | **Major resection**  **(n = 646)** | **Minor resection**  **(n = 919)** | ***P*** |
| --- | --- | --- | --- | --- |
| Age, years | 58.3 ± 9.9 | 57.8 ± 10.0 | 58.6 ± 9.8 | 0.10 |
| Gender,  male/female (%) | 1258/307  (80.4/19.6) | 522/124  (80.8/19.2) | 736/183  (80.1/19.9) | 0.77 |
| Etiologies, n (%)  Alcohol  HBV  HCV  HBV+HCV  NBNC | 101 (6.5)  1,265 (80.8)  52 (3.3)  9 (0.6)  138 (8.8) | 43(6.7)  517 (80.0)  16 (2.5)  3 (0.5)  67 (10.4) | 58 (6.3)  748 (81.4)  36 (3.9)  6 (0.7)  71 (7.7) | 0.22 |
| ASA fitness grade, n (%)  1  2  3  4 | 30 (1.9)  1,385 (88.5)  146 (9.3)  4 (0.3) | 15 (2.3)  565 (87.5)  64(9.9)  2 (0.3) | 15 (1.6)  820 (89.2)  82 (8.9)  2 (0.2) | 0.66 |
| Body mass index, kg/m^2^ | 24.2 ± 3.1 | 23.9 ± 2.9 | 24.4 ± 3.1 | <0.001 |
| Comorbidities, n (%)  Diabetes  Hypertension  Cardiovascular  Renal  Oncologic  Respiratory | 314 (20.1)  546 (34.9)  19 (1.2)  13 (0.8)  30 (1.9)  23 (1.5) | 121 (18.7)  221 (34.2)  5 (0.8)  4 (0.6)  8 (1.2)  6 (0.9) | 193 (21.0)  325 (35.4)  14 (1.5)  9 (1.0)  22 (2.4)  17 (1.8) | 0.30  0.68  0.27  0.62  0.15  0.20 |
| Cirrhosis  Varices  Ascites | 517 (37.9)  13 (0.8)  1 (0.1) | 162 (25.1)  4 (0.6)  0 (0.0) | 308 (33.5)  9 (1.0)  1 (0.1) | <0.001  0.62  0.99 |
| Previous TACE, n (%)  Previous PVE, n (%) | 258 (16.5)  156 (10.0) | 186 (28.8)  148 (22.9) | 72 (7.8)  8 (0.9) | <0.001  <0.001 |
| Baseline laboratory exam  Hemoglobin, g/dL  Platelets, x1,000/mm^3^  Prothrombin time, INR  Creatinine, mg/dL  Albumin, g/dL  AST, IU/L  ALT, IU/L  Total bilirubin, mg/dL  Direct bilirubin, mg/dL  Sodium, mmol/L  Estimated GFR  Alpha-fetoprotein, ng/mL median [IQR]  ICG R15, median [IQR] | 13.6 ± 1.6  178.2 ± 73.8  1.1 ± 0.1  0.9 ± 0.4  3.7 ± 0.4  37.8 ± 31.8  35.0 ± 36.4  0.6 ± 0.4  0.3 ± 0.2  139.9 ± 2.5  92.7 ± 15.4  10.8 [3.6–177.7]  13.2 [10.3–16.6] | 13.4 ± 1.6  191.2 ± 80.1  1.1 ± 0.1  0.9 ± 0.4  3.6 ± 0.4  43.3 ± 33.8  37.0 ± 37.1  0.6 ± 0.4  0.3 ± 0.3  139.6 ± 2.5  93.6 ± 15.3  16.4 [3.8–408.2]  12.8 [10.1–16.1] | 13.8 ± 1.6  169.0 ± 67.5  1.1 ± 0.1  0.9 ± 0.4  3.8 ± 0.4  33.9 ± 29.8  33.6 ± 35.9  0.6 ± 0.4  0.3 ± 0.1  140.2 ± 2.4  92.1 ± 15.4  8.8 [3.4–106.3]  13.2 [10.5–16.8] | <0.001  <0.001  0.58  0.61  <0.001  <0.001  0.06  <0.89  0.02  <0.001  0.06  <0.001  0.03 |
| Operation  Anesthesia time, min, median [IQR]  Operation time, min, median [IQR] | 245 [210–300]  211 [175–263] | 270 [230–322]  232 [194–284] | 230 [200–280]  197 [165–242] | <0.001  <0.001 |
| Abbreviations: ALT: alanine aminotransferase, ASA: American Society of Anesthesiologists, AST: aspartate aminotransferase, GFR: glomerular filtration rate, HBV: hepatitis B virus, HCV: hepatitis C virus, ICG: indocyanine green, INR: international normalized ratio, IQR: interquartile range, NBNC: non-HBV and non-HCV, PVE: portal vein embolization, TACE: transarterial chemoembolization. | | | | |
