# Supplementary material for: A simple and clinically applicable model to predict liver-related morbidity after hepatic resection for hepatocellular carcinoma
Source: PLoS One. 2020 Nov 5;15(11):e0241808. doi: 10.1371/journal.pone.0241808 (PMC7643950; doi:10.1371/journal.pone.0241808)
Supplement: S2 Table — (DOCX) [file pone.0241808.s003.docx]

**S2 Table.** Details of treatment of the 1,565 patients enrolled in this study

| **Resection type** | **Patients (n = 1,565)** |
| --- | --- |
| Major resection | 646 (41.3%) |
| Minor resection | 919 (58.7%) |
| Right hepatectomy  Right hepatectomy ± segment I  Extended right hepatectomy  Right anterior sectionectomy ± segment I  Right posterior sectionectomy ± segment I | 321 (20.5%)  8 (0.6%)  228 (14.6%)  235 (15.0%) |
| Left hepatectomy  Left hepatectomy ± segment I  Extended left hepatectomy  Left lateral sectionectomy ± segment I | 155 (9.9%)  5 (0.2%)  111 (7.1%) |
| Segmentectomy (one segment) | 272 (17.4%) |
| Bisegmentectomy | 85 (5.4%) |
| Resection of three segments | 122 (7.8%) |
| Resection of four segments | 23 (1.5%) |
