# Supplementary material for: A simple and clinically applicable model to predict liver-related morbidity after hepatic resection for hepatocellular carcinoma
Source: PLoS One. 2020 Nov 5;15(11):e0241808. doi: 10.1371/journal.pone.0241808 (PMC7643950; doi:10.1371/journal.pone.0241808)
Supplement: S3 Table — (DOCX) [file pone.0241808.s004.docx]

**S3 Table.** Summary of patients who died after liver resection

| Patient | Age | Sex | Etiology | Cirrhosis | Comorbidity | Type of resection | PT (INR) | Total  bilirubin (mg/dL) | Albumin (g/dL) | Platelet, (mm^3^) | ICG | Time until death, days | Cause of death |
| --- | --- | --- | --- | --- | --- | --- | --- | --- | --- | --- | --- | --- | --- |
| 1 | 51 | M | Alcoholic | 0 |  | Total hepatectomy | 1.26 | 1.0 | 3.2 | 359 | 11.781 | 2 | Massive bleeding, DIC |
| 2 | 74 | M | NBNC | 1 | HTN, COPD | Right ant sectionectomy | 1.05 | 0.5 | 3.4 | 253 | 16.318 | 9 | Septic shock |
| 3 | 46 | M | HCV | 0 | HTN | Right hepatectomy | 1.01 | 0.3 | 3.5 | 153 | 14.166 | 11 | Septic shock |
| 4 | 56 | M | HBV | 1 |  | Right ant sectionectomy | 1.13 | 1.1 | 4.1 | 170 | 12.094 | 24 | Postop bleeding |
| 5 | 47 | M | HBV | 1 |  | Resection of three segments | 0.99 | 0.8 | 3.2 | 151 | NA | 40 | Cardiac tamponade |
| 6 | 67 | M | Alcoholic | 0 | DM, HTN, CKD, Asthma | Segmentectomy | 1.08 | 0.4 | 3.1 | 403 | 22.16 | 40 | Pneumonia |
| 7 | 64 | M | HBV | 1 | HTN | Ext. right hepatectomy | 1.13 | 0.8 | 3 | 280 | 14.993 | 52 | Liver failure |
| 8 | 76 | F | HBV | 1 | HTN | Left lateral sectionectomy | 1.14 | 0.6 | 3.5 | 135 | 12.18 | 61 | Liver failure |
| 9 | 65 | M | HBV | 0 | DM, HTN | Right post sectionectomy | 1.03 | 0.5 | 2.5 | 241 | 26.828 | 65 | Unknown |
| 10 | 41 | M | HBV | 0 |  | Resection of four segments | 1.09 | 0.5 | 4.2 | 189 | 11.315 | 82 | HCC |
| 11 | 61 | M | HBV | 1 | HTN | Right hepatectomy | 1.05 | 0.3 | 3.5 | 89 | 11.669 | 82 | Liver failure |
| 12 | 54 | M | HBV | 1 | DM | Right hepatectomy | 1.28 | 0.6 | 2.7 | 353 | 3.754 | 85 | HCC |
| 13 | 40 | M | HBV | 1 |  | Resection of three segments | 1.18 | 1.0 | 3.6 | 167 | 11.224 | 86 | HCC |
| *Abbreviations: CKD: chronic kidney disease, COPD: chronic obstructive pulmonary disease, DM: diabetes mellitus, HBV: hepatitis B virus, HCV: hepatitis C virus, HTN: hypertension, ICG: indocyanine green, INR: international normalized ratio. | | | | | | | | | | | | | |
