# Supplementary material for: A simple and clinically applicable model to predict liver-related morbidity after hepatic resection for hepatocellular carcinoma
Source: PLoS One. 2020 Nov 5;15(11):e0241808. doi: 10.1371/journal.pone.0241808 (PMC7643950; doi:10.1371/journal.pone.0241808)
Supplement: S4 Table — (DOCX) [file pone.0241808.s005.docx]

**S4 Table.** Comparison of baseline characteristics of patients who did and did not die within 90 days after liver resection for hepatocellular carcinoma

| **Characteristics** | **No mortality within 90 days**  **(N = 1,552)** | **Mortality within 90 days**  **(N = 13)** | ***P*-value** |
| --- | --- | --- | --- |
| Age, years | 58.3 ± 9.9 | 57.1 ± 11.9 | 0.68 |
| Gender, male/female (%) | 1246/306 (80.3/19.7) | 12/1 (92.3/7.7) | 0.46 |
| Etiologies, *n* (%)  Alcohol  HBV  HCV  HBV+HCV  NBNC | 99 (6.4)  1,256 (80.9)  9 (0.6)  51 (3.3)  137 (8.8) | 2 (15.4)  9 (69.2)  0 (0.0)  1 (7.7)  1(7.7) | 0.61 |
| ASA fitness grade, *n* (%)  1  2  3  4 | 30 (1.9)  1,373 (88.5)  145 (9.3)  4 (0.3) | 0 (0.0)  12 (92.3)  1 (7.7)  0 (0.0) | 0.95 |
| Body mass index, *kg/m^2^* | 24.2 ± 3.1 | 24.6 ± 4.8 | 0.79 |
| Comorbidities, *n* (%)  Diabetes  Hypertension  Cardiovascular  Renal  Oncologic  Respiratory | 311 (20.0)  539 (34.7)  19 (1.2)  12 (0.8)  29 (1.9)  21 (1.4) | 3 (23.1)  7 (53.8)  0 (0.0)  1 (7.7)  1 (7.7)  2 (15.4) | 0.99  0.25  0.99  0.23  0.61  0.002 |
| Cirrhosis at pathologic exam  Varices  Ascites | 595 (38.3)  13 (0.8)  1 (0.1) | 8 (61.5)  0 (0.0)  0 (0.0) | 0.15  0.99  0.99 |
| Previous TACE, *n* (%)  Previous PVE, *n* (%) | 256 (16.5)  153 (9.9) | 2 (15.4)  3 (23.1) | 0.99  0.26 |
| Baseline laboratory exam  Hemoglobin  Platelets  Prothrombin time, INR  Creatinine  Albumin  AST  ALT  Total bilirubin  Direct bilirubin  Estimated GFR  Alpha-fetoprotein, *median [IQR]*  ICG R15, *median [IQR]* | 13.7 [12.7–14.7]  167 [131–209]  1.1 [1.0–1.1]  0.8 [0.7–0.9]  3.7 [3.5–4.0]  30 [23–41]  27 [18–40]  0.6 [0.4–0.8]  0.2 [0.2–0.3]  95.0 [86.0–102.0]  10.8 [3.6–175.4]  13.1 [10.2–16.6] | 14.0 [13.5–15.7]  189 [153–280]  1.1 [1.0–1.1]  0.9 [0.8–0.9]  3.4 [3.1–3.5]  44 [25–72]  23 [18–38]  0.6 [0.5–0.8]  0.2 [0.2–0.4]  96.0 [89.0–107.0]  252.6 [4.0–28356.5]  12.1 [11.5–15.7] | 0.31  0.07  0.26  0.99  0.003  0.08  0.77  0.63  0.46  0.64  0.11  0.90 |
| Operation  Anesthesia time, min, median [IQR]  Operation time, min, median [IQR] | 245 [210–300]  211 [175–263] | 268 [245–313]  233 [220–285] | 0.07  0.05 |
| *Abbreviations: ALT: alanine aminotransferase, ASA: American Society of Anesthesiologists physical status, AST: aspartate aminotransferase, GFR: glomerular filtration rate, HBV: hepatitis B virus, HCV: hepatitis C virus, ICG: indocyanine green, INR: international normalized ratio, IQR: interquartile range, NBNC: non-HBV and non-HCV, PVE: portal vein embolization, TACE: transarterial chemoembolization. | | | |
