# Supplementary material for: A simple and clinically applicable model to predict liver-related morbidity after hepatic resection for hepatocellular carcinoma
Source: PLoS One. 2020 Nov 5;15(11):e0241808. doi: 10.1371/journal.pone.0241808 (PMC7643950; doi:10.1371/journal.pone.0241808)
Supplement: S5 Table — (DOCX) [file pone.0241808.s006.docx]

**S5 Table.** Morbidity and mortality rates after liver resection according to baseline characteristics and type of resection

| **Variables** | **Total** | | | **Major resection** | | | **Minor resection** | | |
| --- | --- | --- | --- | --- | --- | --- | --- | --- | --- |
|  | **n** | **Morbidity** | **Mortality** | **n** | **Morbidity** | **Mortality** | **n** | **Morbidity** | **Mortality** |
| Age, <50 years | 294 | 23  (7.8%) | 4  (1.4%) | 133 | 16  (12.0%) | 4  (3.0%) | 161 | 7  (4.3%) | 0  (0.0%) |
| Age, 50–59 years | 543 | 46  (8.5%) | 3  (0.6%) | 223 | 25  (11.2%) | 2  (0.9%) | 320 | 21  (6.6%) | 1  (0.3%) |
| Age, ≥60 years | 728 | 64  (8.8%) | 6  (0.8%) | 290 | 36  (12.4%) | 3  (1.0%) | 438 | 28  (6.4%) | 3  (0.7%) |
| Platelets, <100,000/mm^3^ | 134 | 18  (13.4%) | 1  (0.7%) | 36 | 7  (19.4%) | 1  (2.8%) | 98 | 11  (11.2%) | 0  (0.0%) |
| Platelets, <150,000/mm^3^ | 449 | 48  (10.7%) | 1  (0.2%) | 158 | 28/158  (17.7%) | 1  (0.6%) | 291 | 20  (6.9%) | 0  (0.0%) |
| Platelets, ≥150,000/mm^3^ | 982 | 67  (6.8%) | 11  (1.1%) | 452 | 42/452  (9.3%) | 7  (1.5%) | 530 | 25  (4.7%) | 4  (0.8%) |
| PT (INR), <1.0 | 150 | 6  (4.0%) | 1  (0.7%) | 65 | 4/65  (6.2%) | 1  (1.5%) | 85 | 2  (2.4%) | 0  (0.0%) |
| PT (INR), 1.0–1.1 | 827 | 51  (6.2%) | 6  (0.7%) | 337 | 30/337  (8.9%) | 3  (0.9%) | 490 | 21  (4.3%) | 3  (0.6%) |
| PT (INR), ≥1.1 | 588 | 76  (12.9%) | 6  (1.0%) | 244 | 43/244  (17.6%) | 5  (2.0%) | 344 | 33  (9.6%) | 1  (0.3%) |
| Albumin, <3.5g/dL | 365 | 58  (15.9%) | 7  (1.9%) | 200 | 34/200  (17.0%) | 4  (2.0%) | 165 | 24  (14.5%) | 3  (1.8%) |
| Albumin, 3.5–3.9g/dL | 782 | 51  (6.5%) | 4  (0.5%) | 311 | 33/311  (10.6%) | 4  (1.3%) | 471 | 18  (3.8%) | 0  (0.0%) |
| Albumin, ≥4.0g/dL | 418 | 24  (5.7%) | 2  (0.5%) | 135 | 10/135  (7.4%) | 1  (0.7%) | 283 | 14  (4.9%) | 1  (0.4%) |
| ICG R15, <10% | 363 | 20  (5.5%) | 1  (0.3%) | 157 | 11/157  (7.0%) | 1  (0.6%) | 206 | 9  (4.4%) | 0  (0.0%) |
| ICG R15, <15% | 645 | 49  (7.6%) | 8  (1.2%) | 274 | 30/274  (10.9%) | 7  (2.6%) | 371 | 19  (5.1%) | 1  (0.3%) |
| ICG R15, ≥15% | 557 | 64  (11.5%) | 4  (0.7%) | 215 | 36/215  (16.7%) | 1  (0.5%) | 342 | 28  (8.2%) | 3  (0.9%) |
